# Bivariate quantitative Bayesian LASSO for detecting association of rare haplotypes with two correlated continuous phenotypes

**DOI:** 10.3389/fgene.2023.1104727

**Published:** 2023-03-09

**Authors:** Ibrahim Hossain Sajal, Swati Biswas

**Affiliations:** Department of Mathematical Sciences, University of Texas at Dallas, Richardson, TX, United States

**Keywords:** diastolic blood pressure (DBP), Genetic Analysis Workshop 19, Markov chain Monte Carlo (MCMC), regularization, systolic blood pressure (SBP)

## Abstract

In genetic association studies, the multivariate analysis of correlated phenotypes offers statistical and biological advantages compared to analyzing one phenotype at a time. The joint analysis utilizes additional information contained in the correlation and avoids multiple testing. It also provides an opportunity to investigate and understand shared genetic mechanisms of multiple phenotypes. Bivariate logistic Bayesian LASSO (LBL) was proposed earlier to detect rare haplotypes associated with two binary phenotypes or one binary and one continuous phenotype jointly. There is currently no haplotype association test available that can handle multiple continuous phenotypes. In this study, by employing the framework of bivariate LBL, we propose bivariate quantitative Bayesian LASSO (QBL) to detect rare haplotypes associated with two continuous phenotypes. Bivariate QBL removes unassociated haplotypes by regularizing the regression coefficients and utilizing a latent variable to model correlation between two phenotypes. We carry out extensive simulations to investigate the performance of bivariate QBL and compare it with that of a standard (univariate) haplotype association test, Haplo.score (applied twice to two phenotypes individually). Bivariate QBL performs better than Haplo.score in all simulations with varying degrees of power gain. We analyze Genetic Analysis Workshop 19 exome sequencing data on systolic and diastolic blood pressures and detect several rare haplotypes associated with the two phenotypes.

## 1 Introduction

Information on multiple phenotypes is often collected in health-related studies to obtain a bigger picture of patients’ health conditions (Teixeira-Pinto and Normand, 2009). Studies have found variants at numerous genetic loci to be associated with these phenotypes (Solovieff et al., 2013). Sometimes, a genetic variant is associated with more than one phenotype, a phenomenon known as pleiotropy. Recent studies have confirmed the widespread presence of pleiotropy in the human genome, thus showing the underlying common genetic mechanisms of numerous traits (Solovieff et al., 2013; Gratten and Visscher, 2016; Buniello et al., 2019; Watanabe et al., 2019). Investigating and understanding pleiotropy can uncover additional associations, redefine disease classification, and expand our understanding of the genetic basis of complex diseases with wide-ranging implications for healthcare (Hackinger and Zeggini, 2017; Lee et al., 2019; Lee et al., 2021).

The most common way of the testing trait–variant association is to consider one phenotypic trait at a time and test its association with genotypic variants under study. However, such a univariate statistical approach ignores valuable additional information contained in the joint distribution of the phenotypes. Even more importantly, such an approach amounts to a lost opportunity to investigate potential pleiotropy and shared genetic mechanisms. It may also result in a loss of power, especially with multiplicity adjustment, for performing multiple univariate tests. Therefore, considering a multivariate framework to model the phenotypes jointly is appealing from both biological and statistical perspectives.

Several methods have been proposed that utilize a multivariate framework to jointly model multiple correlated phenotypes, including some recent gene-based approaches (Klei et al., 2008; O’Reilly et al., 2012; Van der Sluis et al., 2015; Ray et al., 2016; Hackinger and Zeggini, 2017; Kaakinen et al., 2017; Lee et al., 2017; Ray and Basu, 2017; Deng et al., 2020). However, most of these studies consider single-nucleotide polymorphisms (SNPs) or variants (SNVs) as a genetic unit obtained from genome-wide association studies (GWAS) or next-generation sequencing (NGS) studies. Thus, when rare variants are of interest, one has to rely on SNVs obtained from NGS as rare SNPs are not usually genotyped in GWAS. Yet, most NGS data lack the adequate sample size required for multivariate analysis of correlated phenotypes. Hence, an alternative approach to multiple trait–rare variant association tests that does not necessarily rely on NGS data is warranted.

Haplotype-based tests are powerful alternatives to SNP-based genetic association tests (Bader, 2001; Wang and Lin, 2015). Haplotypes are more biologically meaningful genetic variants as compared to SNPs, which are not inherited independently. Moreover, common SNPs can make up a rare haplotype in a haplotype block, providing avenues to investigate the common disease rare variant (CDRV) hypothesis. Thus, rare variants can also be investigated using GWAS data through haplotype-based tests, allowing the use of data from much larger sample sizes than those of NGS. Several tests have been proposed to investigate the CDRV hypothesis through haplotype-based tests (Guo and Lin, 2009; Li et al., 2010; Li et al., 2011; Biswas and Lin, 2012; Lin et al., 2013), among which logistic Bayesian LASSO (LBL) is a well-studied and powerful method (Biswas and Lin, 2012; Biswas and Papachristou, 2014; Datta and Biswas, 2016; Papachristou and Biswas, 2020). LBL was extended to incorporate gene–environment interactions (Zhang et al., 2017a; Zhang et al., 2017b; Papachristou and Biswas, 2020), data generated using complex sampling designs (Zhang et al., 2017a), and family data (Wang and Lin, 2014; Datta et al., 2018). LBL was also adapted to accommodate two phenotypes, namely, bivariate LBL-2B for binary phenotypes and bivariate LBL-BC for binary and continuous phenotypes (Yuan and Biswas, 2019; Yuan and Biswas, 2021). LBL and its extensions utilize regularization to decrease the unassociated effects close to zero, which, in turn, helps the effect of an associated haplotype, especially if it is a rare one, to stand out. Bivariate LBL-2B and LBL-BC model the dependency between two phenotypes *via* a latent variable. Notably, there is another haplotype-based bivariate genetic association test for correlated quantitative traits; it uses the haplotype trend regression approach (Pei et al., 2009). However, it is only applicable for testing associations with common haplotypes and hence cannot be used for the CDRV hypothesis.

There is no haplotype-based association test currently available that can detect rare haplotypes associated with multiple quantitative phenotypes jointly. To fill this gap, we propose a new method, bivariate quantitative Bayesian LASSO (QBL) to jointly model two correlated continuous phenotypes. We borrow the well-studied framework of bivariate LBL and make appropriate modifications to accommodate quantitative traits. The properties of bivariate QBL are investigated using extensive simulations under various association scenarios, sample sizes, and the number of haplotypes. We also compare its performance to a standard univariate haplotype-based association test, Haplo.score (Schaid et al., 2002). Finally, we apply our proposed method to exome sequencing data from Genetic Analysis Workshop (GAW) 19. We analyze haplotype blocks in several genes of interest (as per literature) and detect rare haplotypes associated with systolic and diastolic blood pressures (SBP and DBP) jointly.

## 2 Methods

### 2.1 Likelihood formulation

We closely follow the framework of bivariate LBL-2B and LBL-BC and accordingly the notations used therein. Consider a sample of n subjects with two continuous correlated (standardized) phenotypes denoted by $Y_{ic}$ and $Y_{ic^{\prime }}$ . Let $Y_{c}=\left(Y_{1c,}Y_{2c,\ldots ,}Y_{nc}\right)$ , $Y_{c^{\prime }}=\left(Y_{1c^{\prime },}Y_{2c^{\prime },\ldots ,}Y_{nc^{\prime }}\right)$ , and $G=\left(G_{1},G_{2},\ldots ,G_{n}\right)$ , where $G_{i}$ represents the $i^{th}$ individual’s observed genotype on the SNPs, making up the haplotype block under study. Furthermore, let $S\left(G_{i}\right)$ be the set of haplotype pairs compatible with $G_{i}$ as the haplotype pair for an individual may not be unambiguously determined from the genotype data; $Z_{ir}$ denotes the $r^{th}$ element of $S\left(G_{i}\right)$ . We introduce a latent variable $u_{i}$ to model the marginal dependence between $Y_{ic}$ and $Y_{ic^{\prime }}$ . Let $u_{i}\;∼\;N\left(0,\sigma_{u}^{2}\right)$ for all i and $u=\left(u_{1},u_{2}{,}_{\ldots ,}u_{n}\right)$ . We assume that although $Y_{ic}$ and $Y_{ic^{\prime }}$ are marginally dependent, they are conditionally independent, given $u_{i}$ . In other words, the latent variable induces conditional independence between the two correlated outcomes. We also assume that $Z_{ir}$ is independent of $u_{i}$ . The likelihood can be written as

$$L\left(\psi \right)=\overset{n}{\underset{i=1}{\prod }}\underset{Z_{ir}\epsilon S\left(G_{i}\right)}{\sum }P\left(Y_{ic},Y_{ic^{\prime }},Z_{ir},u_{i}\right)$$

$$\propto \overset{n}{\underset{i=1}{\prod }}\underset{Z_{ir}\epsilon S\left(G_{i}\right)}{\sum }P\left(Y_{ic},Y_{ic^{\prime }}|Z_{ir},u_{i}\right)P\left(Z_{ir},u_{i}\right)$$

$$\propto \overset{n}{\underset{i=1}{\prod }}\underset{Z_{ir}\epsilon S\left(G_{i}\right)}{\sum }P\left(Y_{ic}|Z_{ir},u_{i}\right)P\left(Y_{ic^{\prime }}|Z_{ir},u_{i}\right)P\left(Z_{ir}\right)P\left(u_{i}\right),$$

where ψ is the vector of model parameters, which includes regression coefficients, variance parameters, and parameters associated with haplotype frequencies (to be introduced soon). Notably, bivariate QBL does not require specification of the haplotype pair for an individual (which is typically unknown due to phase ambiguity); rather, it averages over all compatible haplotype pairs for a person to incorporate uncertainty in haplotype pair estimation. Suppose there are m possible haplotypes in the haplotype block and population under study. In the following, we model the probabilities in the aforementioned likelihood in terms of the model parameters (the subscripts i and r are suppressed for simplicity).

#### 2.1.1 Modeling of $P\left(Y_{c}|Z,u\right)$ and $P\left(Y_{c^{\prime }}|Z,u\right)$

A haplotype pair Z consists of two haplotypes denoted as $z_{k}/z_{k^{\prime }}\;\left(k,k^{\prime }=1,2,\ldots ,m\right)$ . Let $X_{z}=\left(1,x_{1},x_{2},\ldots ,x_{m-1}\right)$ be a (row) design vector with $x_{k}$ equal to the number of times $z_{k}$ appears in the haplotype pair Z ; k=1,…,m−1 , i.e., $z_{k}=0,1,$ or 2 . The $m^{th}$ haplotype is assumed to be the baseline without loss of generality. Let $\beta_{c}$ and $\beta_{c^{\prime }}$ be the vectors of regression coefficients (including the intercept), i.e., they include the effects of haplotypes on phenotypes $Y_{c}$ and $Y_{c^{\prime }}$ , respectively. The slope coefficients have the same interpretation as in a usual linear regression model, i.e., the expected change in the quantitative trait if a person carries a copy of a specific haplotype as opposed to the baseline haplotype. As $Y_{c}$ and $Y_{c^{\prime }}$ are two continuous phenotypes and *u* is the latent variable that induces a correlation between them, we use the following linear models: $Y_{c}=X_{z}\beta_{c}+u+\epsilon_{c}$ and $Y_{c^{\prime }}=X_{z}\beta_{c^{\prime }}+u+\epsilon_{c^{\prime }}$ , where $\epsilon_{c}\;∼\;N\left(0,\sigma_{c}^{2}\right)$ and $\epsilon_{c^{\prime }}∼\;N\left(0,\sigma_{c^{\prime }}^{2}\right)$ . We assume $\epsilon_{c},\epsilon_{c^{\prime }}$ , and u to be uncorrelated with each other. The marginal correlation coefficient between $Y_{c}$ and $Y_{c^{\prime }}$ can be shown to be equal to $\frac{\sigma_{u}^{2}}{\sqrt{\sigma_{u}^{2}+\sigma_{c}^{2}}\sqrt{\sigma_{u}^{2}+\sigma_{c^{\prime }}^{2}}}$ and, thus, must be non-negative. If the two traits are negatively correlated, then the values for one of them should be multiplied by −1 before applying this method.

#### 2.1.2 Modeling $P\left(Z\right)$

We model $P\left(Z\right)$ in terms of two sets of parameters: $f=\left(f_{1},f_{2},\ldots ,f_{m}\right),$ denoting the frequencies of m haplotypes in the population, and d , the within-population inbreeding coefficient (Weir, 1996).

For a given haplotype pair $Z=z_{k}/z_{k^{\prime }}$

$$P(Z)=P\left.\left(Z=z_{k}/z_{k^{\prime }}\right.\right|f,d)=\delta_{kk^{\prime }}df_{k}+\left(2-\delta_{kk^{\prime }}\right)\left(1-d\right)f_{k}f_{k^{\prime }}$$

where $\delta_{kk^{\prime }}=1\left(0\right)$ if $z_{k}=z_{k^{\prime }}\left(z_{k}\neq z_{k^{\prime }}\right)$ and $d\in \left(-1,1\right)$ capture the excess/reduction of homozygosity. The aforementioned expression of $P\left(Z\right)$ reduces to the assumption of Hardy–Weinberg equilibrium (HWE) when *d* = 0, while other values of *d* allow for the Hardy–Weinberg disequilibrium.

### 2.2 Prior distributions

There are many choices of shrinkage priors to regularize the regression coefficients, such as LASSO, ridge, Student’s *t*-test, horseshoe, and spike and slab. However, their performances are rather similar when the number of predictors (haplotypes) is smaller than the sample size, as is the case in this study (Van Erp et al., 2019). We choose Bayesian LASSO to regularize the regression coefficients for its ease of implementation, following previous LBL versions. Specifically, the prior for each slope parameter in $\beta_{c}$ and $\beta_{c^{\prime }}$ is assigned a double exponential distribution with mean 0 and variance $\frac{2}{\lambda_{c}^{2}}$ and $\frac{2}{\lambda_{c^{\prime }}^{2}}$ , respectively. We use standard normal priors for the intercepts $\beta_{0c}$ and $\beta_{0c^{\prime }}$ . The amounts of penalty for the slope coefficients are controlled by the hyper-parameters $\lambda_{c}$ and $\lambda_{c^{\prime }}$ . We let them follow gamma (*a*, *b*) distribution with *a* = *b* = 20, following the original LBL method and its extensions (Biswas and Lin, 2012; Yuan and Biswas, 2019; Yuan and Biswas, 2021).

The prior for the frequency vector ***f*** is set to be non-informative Dirichlet (1, …, 1) consisting of *m* values. We consider a uniform prior for *d*. However, given that $P\left(Z\right),$ as shown in Section 2.1.2, must always be non-negative, *d* and ***f*** are not independent. In particular, d must be greater than $-\frac{f_{k}}{1-f_{k}}$ for all *k* values. Thus, the prior for *d*, given ***f*,** is set to be $Uniform\left(\max_{k}\left\{-\frac{f_{k}}{1-f_{k}}\right\},1\right).$ We use a weakly informative half-Cauchy prior for $\sigma_{u}$ with a fixed hyper-parameter *A* given by $\pi \left(\sigma_{u}\right)\propto {\left(1+{\left(\frac{\sigma_{u}}{A}\right)}^{2}\right)}^{-1}$ , where $\sigma_{u}>0$ , and set A=10 (Yuan and Biswas, 2019; Yuan and Biswas, 2021). A non-informative uniform prior is used for $\sigma_{c}^{2}$ and $\sigma_{c^{\prime }}^{2}$ , whose probability density function is given by $p\left(\sigma^{2}\right)\propto \sigma^{-1}$ , where $\sigma^{2}>0$ .

### 2.3 Posterior distributions

The joint posterior distribution of all parameters can be obtained by combining the likelihood and prior distributions as follows:

$$\pi \left(\beta_{c},\beta_{c^{\prime }},\lambda_{b},\lambda_{c},f,d,\sigma_{u},\sigma_{c}^{2},\sigma_{c^{\prime }}^{2},Z|Y_{c},Y_{c^{\prime }},G,u\right)\propto L\left(\Psi \right)\;\pi \left(\beta_{c}|\lambda_{c}\right)\;\pi \left(\beta_{c^{\prime }}|\lambda_{c^{\prime }}\right)\;\pi \left(\lambda_{c}\right)\;\pi \left(\lambda_{c^{\prime }}\right)\;\pi \left(d|f\right)\;\pi \left(f\right)\;\pi \left(\sigma_{u}\right)\;\pi \left(\sigma_{c}^{2}\right)\;\pi \left(\sigma_{c^{\prime }}^{2}\right)$$

where Z consists of all possible haplotype pairs for all n subjects. We use Markov chain Monte Carlo (MCMC) methods to estimate the posterior distributions of all parameters. Details of the MCMC algorithm can be found in Supplementary Appendix A1. Notably, we update the latent variable *u* at every MCMC iteration, and thus, obtain its posterior distribution.

### 2.4 Association testing

We use the posterior distributions of regression coefficients for testing the association of haplotypes with the two phenotypes jointly. In particular, to test the association of the $j^{th}$ haplotype with the two continuous phenotypes jointly, the hypotheses are

$$H_{0}∶\left|\beta_{jc}\right|\leq \epsilon \;and\left|\beta_{jc^{\prime }}\right|\leq \epsilon \;vs\;H_{a}∶\left|\beta_{jc}\right|\;>\epsilon \;or\;\left|\beta_{jc^{\prime }}\right|\;>\epsilon$$

where we set ϵ to be 0.1 (Biswas and Lin, 2012; Yuan and Biswas, 2019; Yuan and Biswas, 2021). Notably, the alternate hypothesis corresponds to the association with at least one phenotype.

To carry out this test, we calculated the Bayes factor (BF), which is the ratio of the posterior odds to the prior odds in favor of the alternative hypothesis. The prior odds can be found in Supplementary Appendix A2.

The posterior odds are obtained from the estimated posterior distributions. Once the BF for each haplotype in a block is obtained, their maximum BF is recorded. If this maximum BF exceeds a certain threshold, we conclude that the haplotype block is associated with at least one of the two phenotypes. We calculated the appropriate threshold following Yuan and Biswas (2019) and Yuan and Biswas (2021)—to be described in detail in the Simulation study and Application sections.

We compare the performance of bivariate QBL with a standard haplotype association test, Haplo.score (Schaid et al., 2002). We use the R package Haplo.stats to apply Haplo.score twice to the two continuous phenotypes individually (Sinnwell and Schaid, 2022).

## 3 Simulation study

### 3.1 Data generation

We generate data under two haplotype settings and five association scenarios to examine the properties of bivariate QBL and compare with Haplo.score. The two haplotype settings consist of 6 and 12 haplotypes (in a haplotype block under this study), as shown in Table 1. Following the simulation studies conducted previously for investigating univariate and bivariate LBL methods, we formed each haplotype by combining five SNPs (to allow easy comparison across various LBL versions). However, we note that, in principle, bivariate QBL can handle haplotype blocks with a larger number of SNPs at the expense of an increased computational burden (this issue is discussed in the Discussion section). Under each setting, the causal haplotype is 11011, a rare haplotype of frequency 1%. This target haplotype can be associated with one or both phenotype(s) and its effect(s), i.e., the corresponding β coefficient(s) can be positive (risk) or negative (protective). This leads to five association scenarios in total with the non-zero β values (for 11011) chosen to ensure that the power of the proposed method or Haplo.score at type I error rates of 0.5%–10% is in a reasonable range. We assume other haplotypes in the block to be null or non-associated, i.e., their β coefficients are equal to 0.

**TABLE 1:** Haplotype settings and association scenarios (the effect of target haplotype is shown in boldface).

Setting	Hap	Freq	Scenario 1		Scenario 2		Scenario 3		Scenario 4		Scenario 5	
			$\beta_{c}$	$\beta_{c^{\prime }}$	$\beta_{c}$	$\beta_{c^{\prime }}$	$\beta_{c}$	$\beta_{c^{\prime }}$	$\beta_{c}$	$\beta_{c^{\prime }}$	$\beta_{c}$	$\beta_{c^{\prime }}$
1	01100	0.300	0	0	0	0	0	0	0	0	0	0
	10100	0.005	0	0	0	0	0	0	0	0	0	0
	11011	0.010	**1**	**1**	**−1**	**−1**	**1**	**−1**	**1**	**0**	**−1**	**0**
	11100	0.155	0	0	0	0	0	0	0	0	0	0
	11111	0.110	0	0	0	0	0	0	0	0	0	0
	10011	0.420	0	0	0	0	0	0	0	0	0	0
2	00111	0.070	0	0	0	0	0	0	0	0	0	0
	01000	0.020	0	0	0	0	0	0	0	0	0	0
	01011	0.050	0	0	0	0	0	0	0	0	0	0
	01101	0.060	0	0	0	0	0	0	0	0	0	0
	01110	0.140	0	0	0	0	0	0	0	0	0	0
	10010	0.080	0	0	0	0	0	0	0	0	0	0
	10100	0.005	0	0	0	0	0	0	0	0	0	0
	11011	0.010	**1**	**1**	**−1**	**−1**	**1**	**−1**	**1**	**0**	**−1**	**0**
	11101	0.090	0	0	0	0	0	0	0	0	0	0
	11110	0.130	0	0	0	0	0	0	0	0	0	0
	11111	0.100	0	0	0	0	0	0	0	0	0	0
	10001	0.245	0	0	0	0	0	0	0	0	0	0

Hap, haplotype; Freq, haplotype frequency.

To generate a haplotype pair for a subject, we use the haplotype frequencies, as shown in Table 1. Using those frequencies and assuming HWE, the probabilities of all possible haplotype pairs can be calculated. Based on those probabilities, we randomly generate one haplotype pair, say *Z*, for each subject in the sample, which corresponds to a design row vector $X_{Z}$ . After assigning haplotype pairs to all subjects, we generate two continuous phenotypes for each subject using the following bivariate normal (BVN) distribution.

$$\left(\begin{array}{c} Y_{c}\\ Y_{c^{\prime }} \end{array}\right)∼\;BVN\left((\begin{array}{c} X_{z}\beta_{c}\\ X_{z}\beta_{c^{\prime }} \end{array}),(\begin{array}{cc} \sigma_{c}^{2} & \rho \sigma_{c}\sigma_{c^{\prime }}\\ \rho \sigma_{c}\sigma_{c^{\prime }} & \sigma_{c^{\prime }}^{2} \end{array})\right),$$

where $\beta_{c}$ and $\beta_{c^{\prime }}$ (excluding intercepts $\beta_{0c}$ and $\beta_{0c^{\prime }}$ ) are as shown in Table 1; $\sigma_{c}=\sigma_{c^{\prime }}=1$ and ρ are varied to be 0.1,0.5, or 0.9 . We set $\beta_{0c}=\beta_{0c^{\prime }}=25$ .

We generate samples of sizes 500 and 1,000. For each sample size and simulation setup, resulting from a combination of a haplotype setting, a non-null association scenario, and a fixed ρ -value, 500 samples are generated. We also generate the corresponding null scenarios, i.e., for each combination of sample size, haplotype setting, and ρ -value, all β s are set to be equal to 0 and 1,000 samples are generated. To each sample, we apply bivariate QBL to both phenotypes jointly. The MCMC is run for a total of 3,00,000 iterations with 50,000 burn-in to achieve acceptable convergence (Gelman et al., 2003). To declare significance, we use appropriate cutoffs to the resulting BFs. The determination of the cutoffs for both bivariate QBL and Haplo.score is discussed in the following sub-section.

### 3.2 Calculation of cutoffs

The cutoffs for bivariate QBL are calculated in the following way. For each sample, we obtain one BF value per haplotype. We record the maximum of those BFs. Thus, we obtain 1,000 maximum BF values from the 1,000 null scenario replicates. We sort these 1,000 values in a descending order and obtain the cutoff for a specific type I error rate to be the corresponding percentile. It is to be noted that by taking the maximum overall BF values from a haplotype block, we adjust for multiple testing within that block.

We calculate cutoffs for Haplo.score in a slightly different way because it is applied to each phenotype. For each sample, we obtain two (global) *p*-values from two Haplo.score analyses. Then, we record the minimum of these two *p*-values. Similar to bivariate QBL, we obtain 1,000 minimum *p*-values from the 1,000 null samples. We sort them in an ascending order and obtain the cutoff of Haplo.score for a specific type I error rate by taking the relevant bottom percentile.

Once the cutoffs are obtained in the aforementioned manner, we use these cutoffs to calculate power for the corresponding non-null setups described previously. The type I error rates and power obtained by varying the cutoffs for a *p*-value (for Haplo.score) and BF (bivariate QBL) are then plotted against each other to obtain receiver operating characteristic (ROC)-type curves. For Haplo.score, the power is shown for detecting associations with at least one of the two phenotypes, as well as with each phenotype separately (in scenarios 1–3, where the target haplotype is associated with both phenotypes).

### 3.3 Results

**FIGURE 1:**
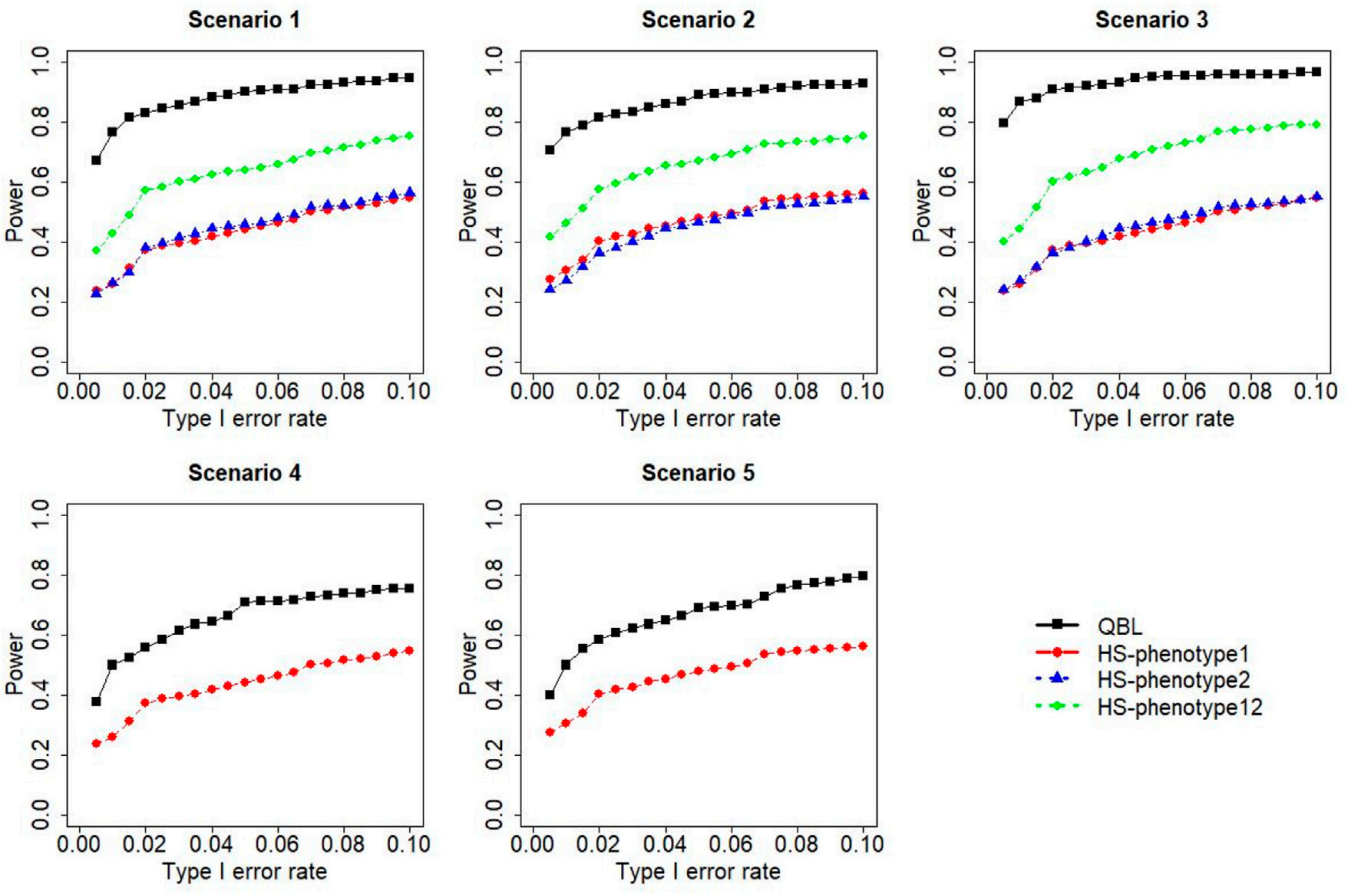
Simulation results under sample size 500, setting 1 (six haplotypes), and *ρ* = 0.1. Scenarios are shown in Table 1. HS, Haplo.score; phenotype12, phenotype 1 or 2.

**FIGURE 2:**
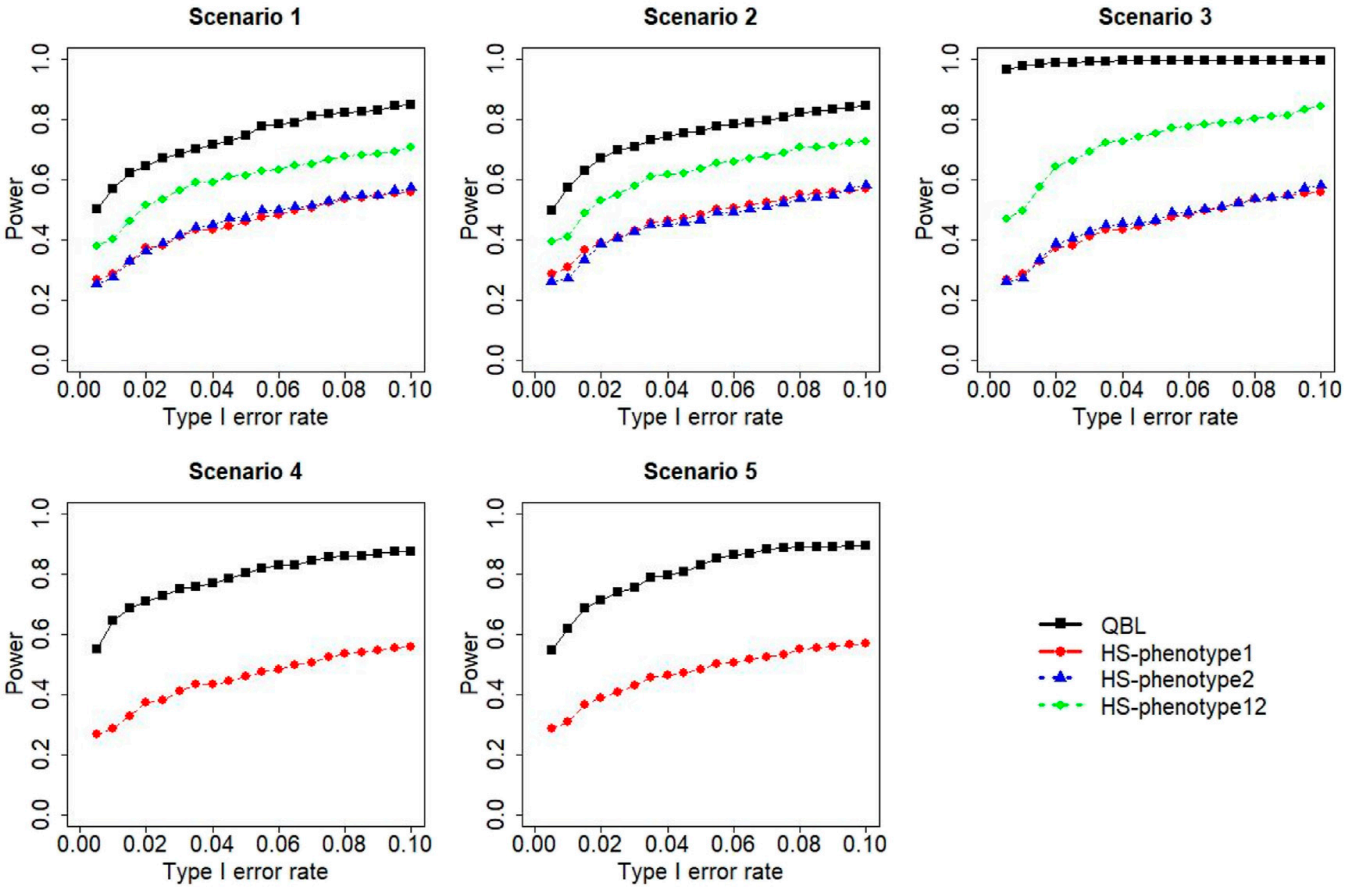
Simulation results under sample size 500, setting 1 (six haplotypes), and *ρ* = 0.5. Scenarios are shown in Table 1. HS, Haplo.score; phenotype12, phenotype 1 or 2.

**FIGURE 3:**
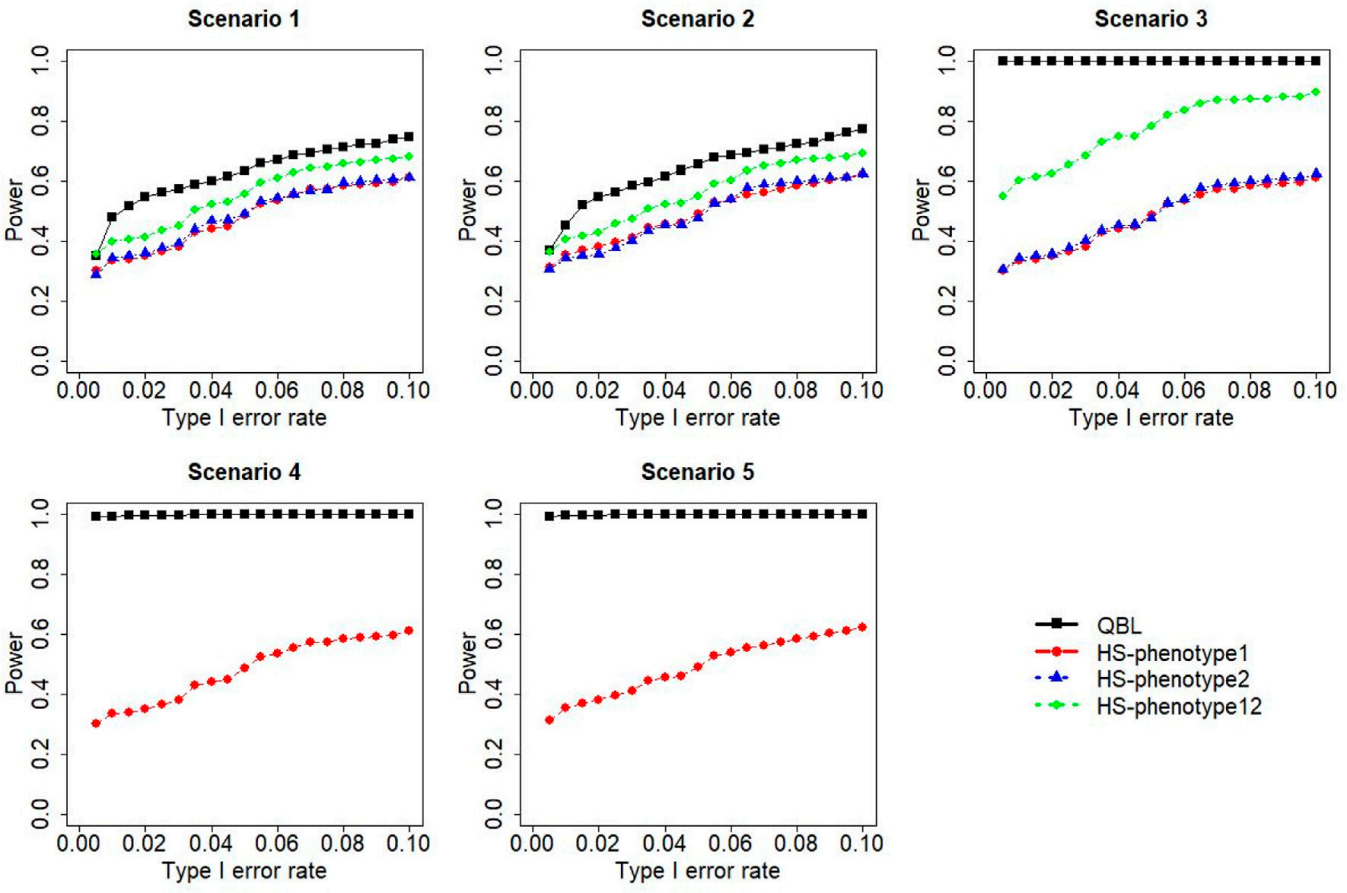
Simulation results under sample size 500, setting 1 (six haplotypes), and *ρ* = 0.9. Scenarios are shown in Table 1. HS, Haplo.score; phenotype12, phenotype 1 or 2.

**FIGURE 4:**
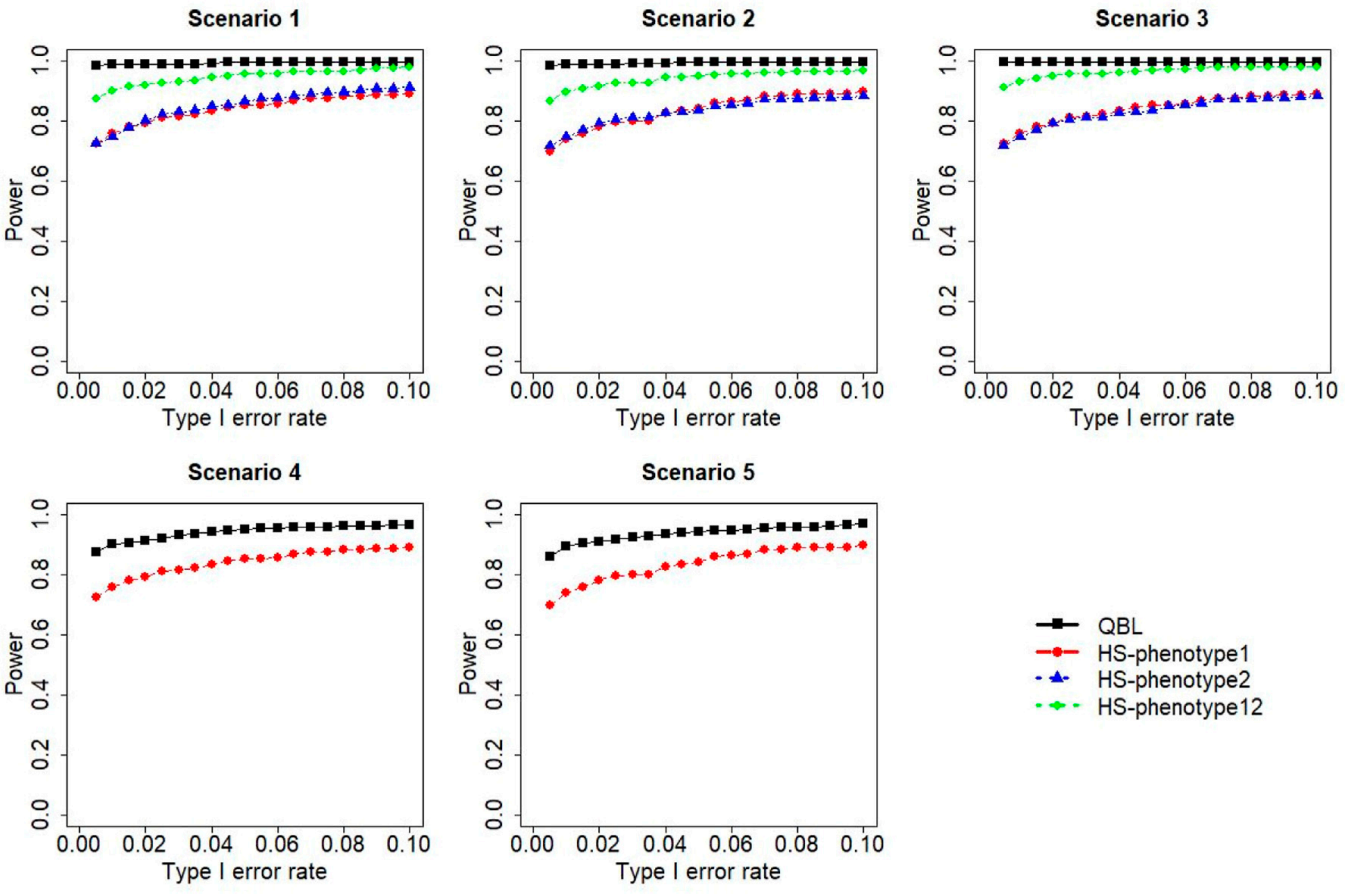
Simulation results under sample size 1,000, setting 1 (six haplotypes), and *ρ* = 0.1. Scenarios are shown in Table 1. HS, Haplo.score; phenotype12, phenotype 1 or 2.

**FIGURE 5:**
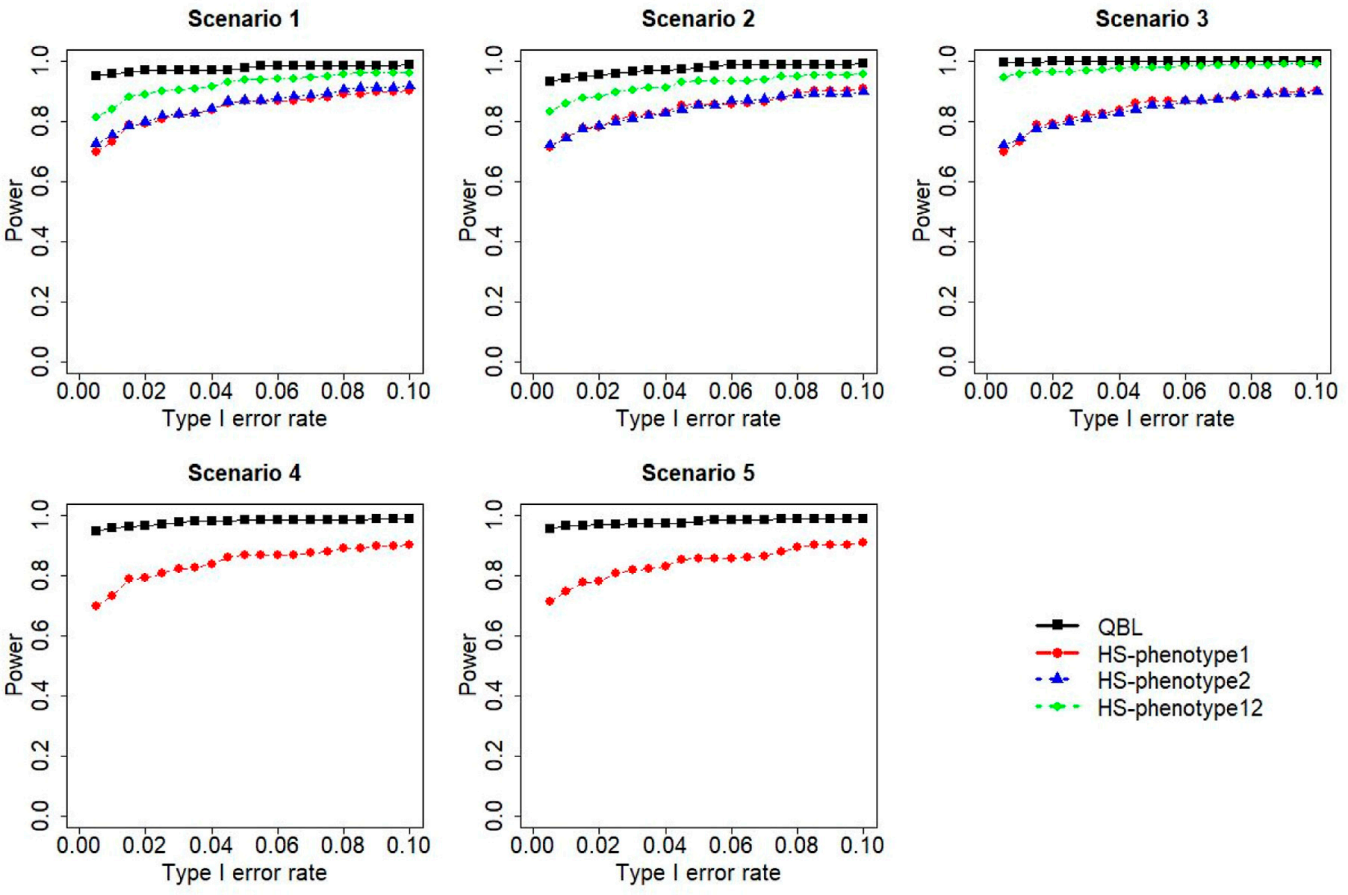
Simulation results under sample size 1,000, setting 1 (six haplotypes), and *ρ* = 0.5. Scenarios are shown in Table 1. HS, Haplo.score; phenotype12, phenotype 1 or 2.

**FIGURE 6:**
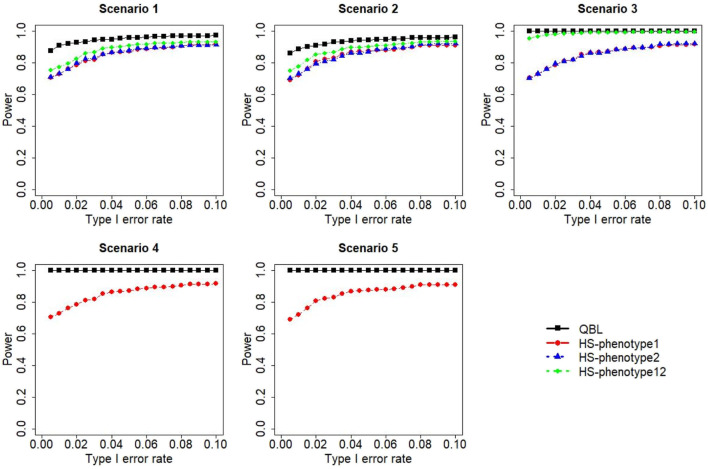
Simulation results under sample size 1,000, setting 1 (six haplotypes), and *ρ* = 0.9. Scenarios are shown in Table 1. HS, Haplo.score; phenotype12, phenotype 1 or 2.

**FIGURE 7:**
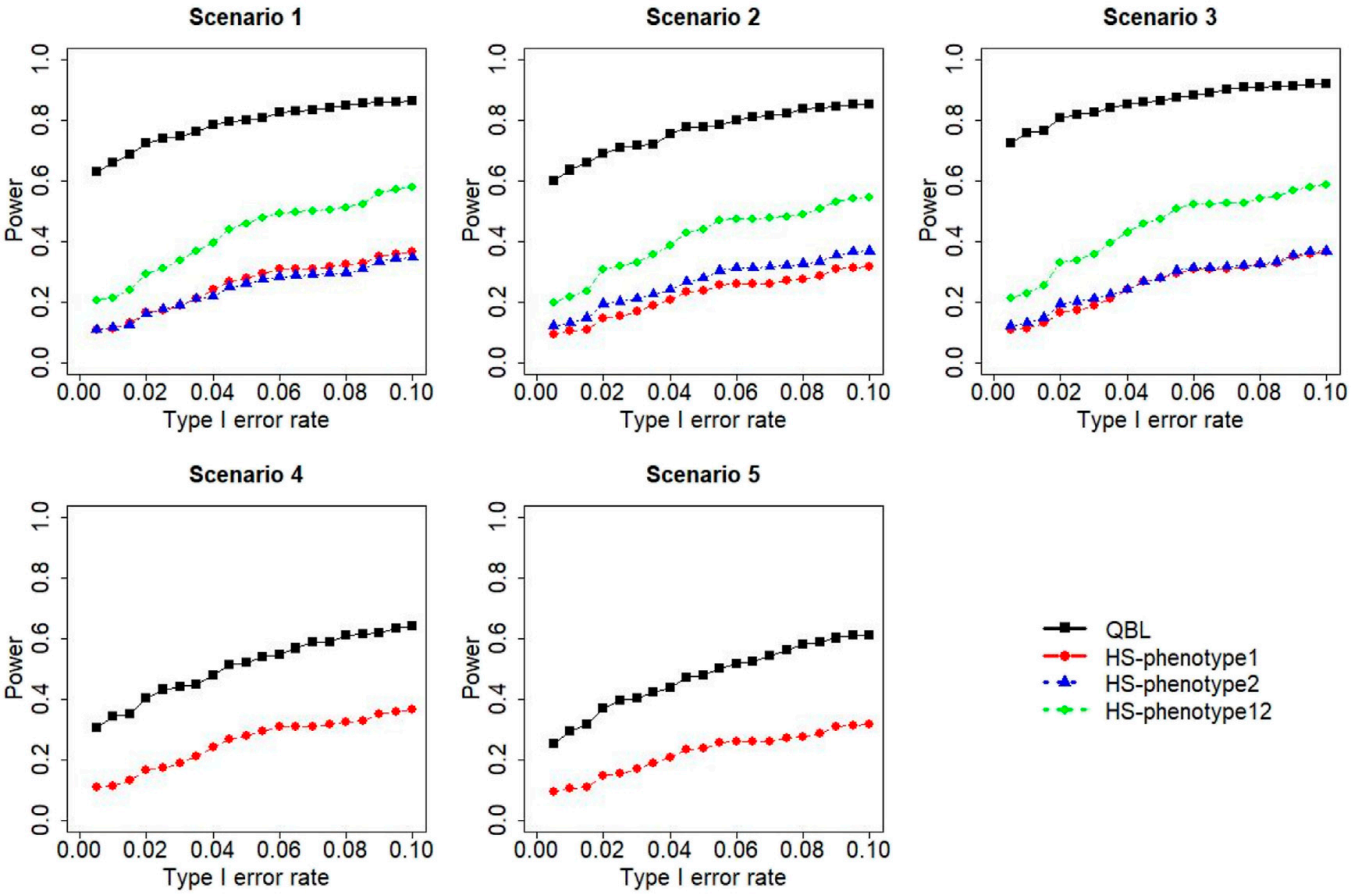
Simulation results under sample size 500, setting 2 (12 haplotypes), and *ρ* = 0.1. Scenarios are shown in Table 1. HS, Haplo.score; phenotype12, phenotype 1 or 2.

**FIGURE 8:**
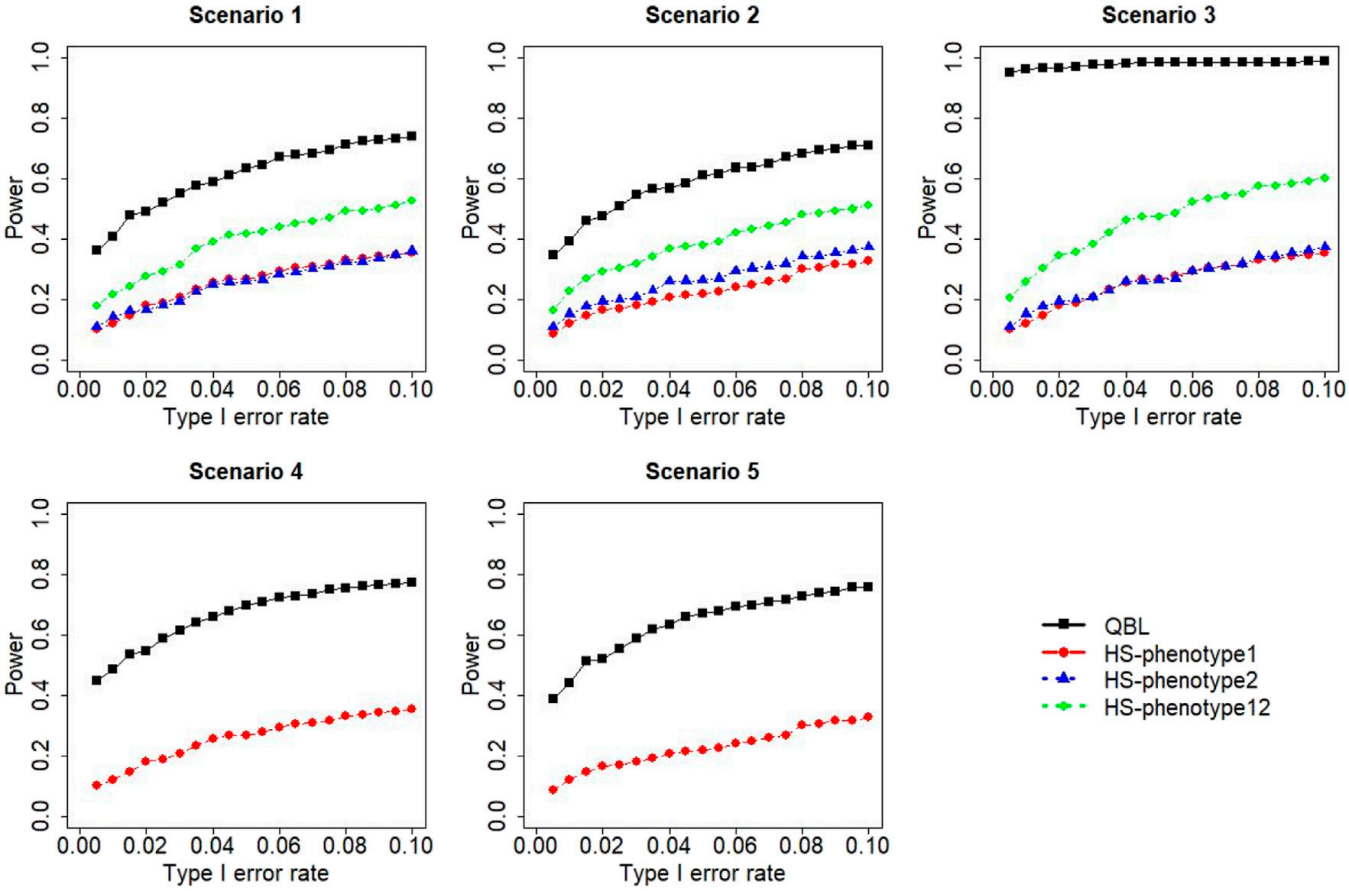
Simulation results under sample size 500, setting 2 (12 haplotypes), and *ρ* = 0.5. Scenarios are shown in Table 1. HS, Haplo.score; phenotype12, phenotype 1 or 2.

**FIGURE 9:**
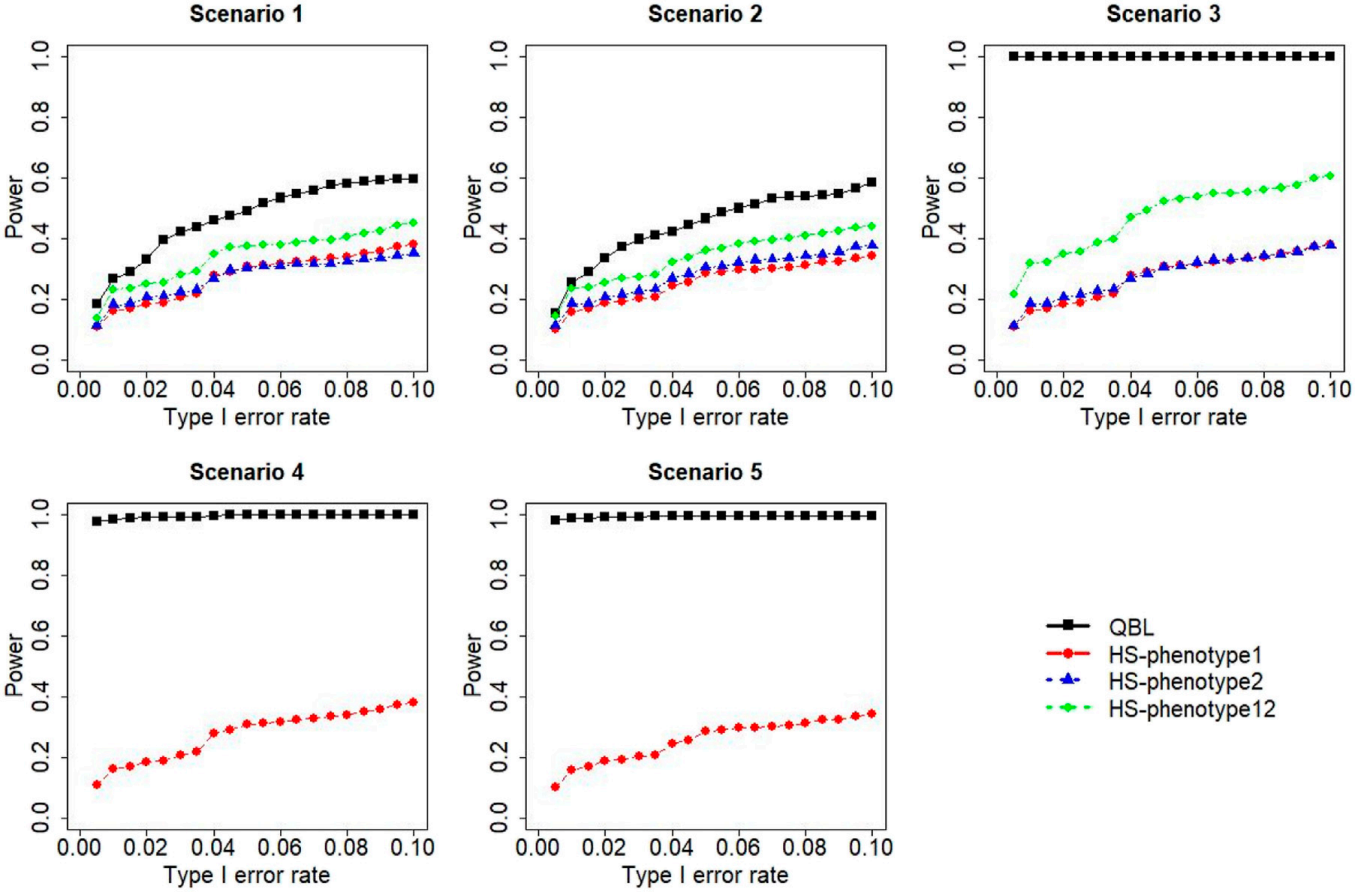
Simulation results under sample size 500, setting 2 (12 haplotypes), and *ρ* = 0.9. Scenarios are shown in Table 1. HS, Haplo.score; phenotype12, phenotype 1 or 2.

**FIGURE 10:**
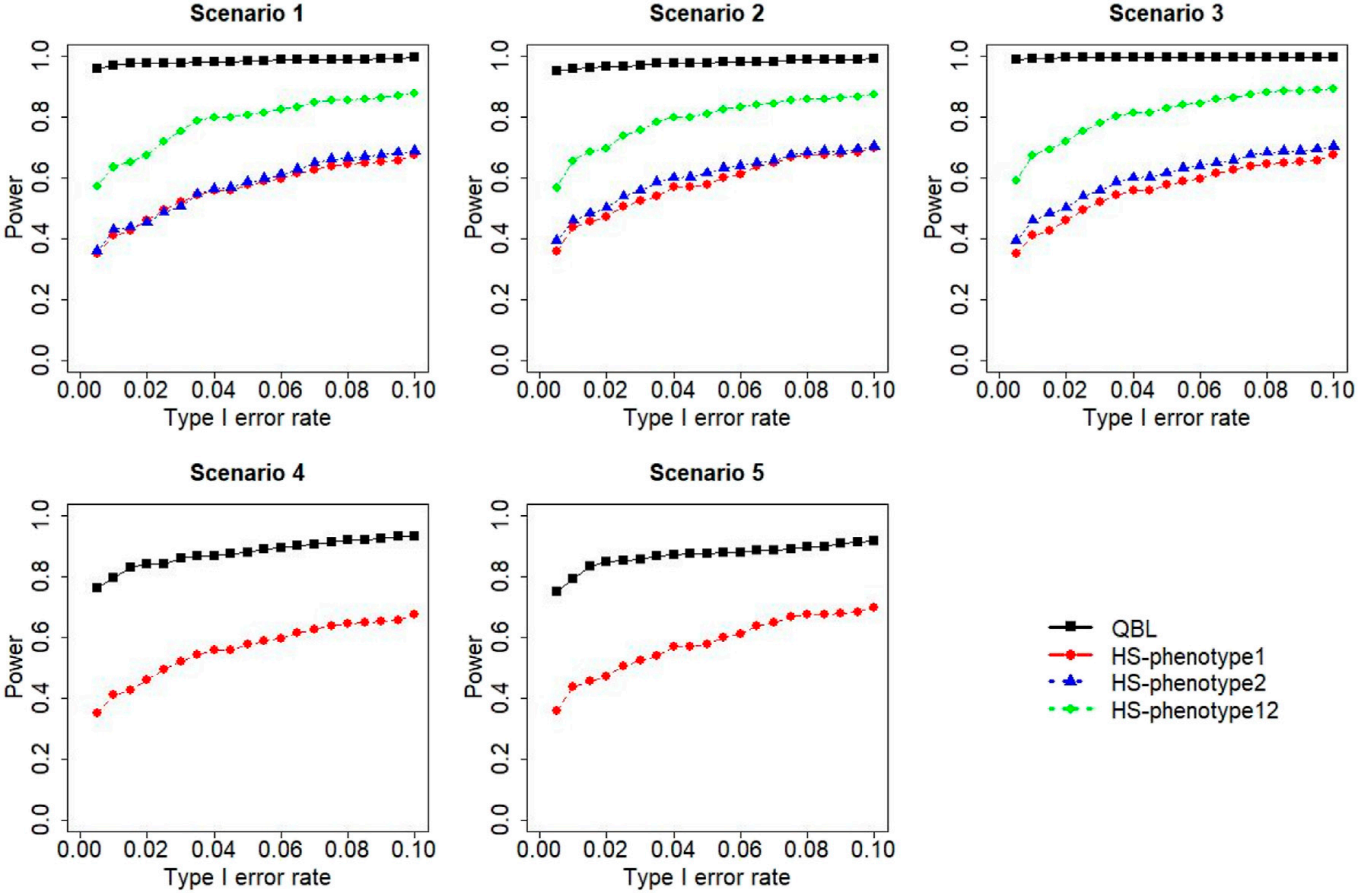
Simulation results under sample size 1,000, setting 2 (12 haplotypes), and *ρ* = 0.1. Scenarios are shown in Table 1. HS, Haplo.score; phenotype12: phenotype 1 or 2.

**FIGURE 11:**
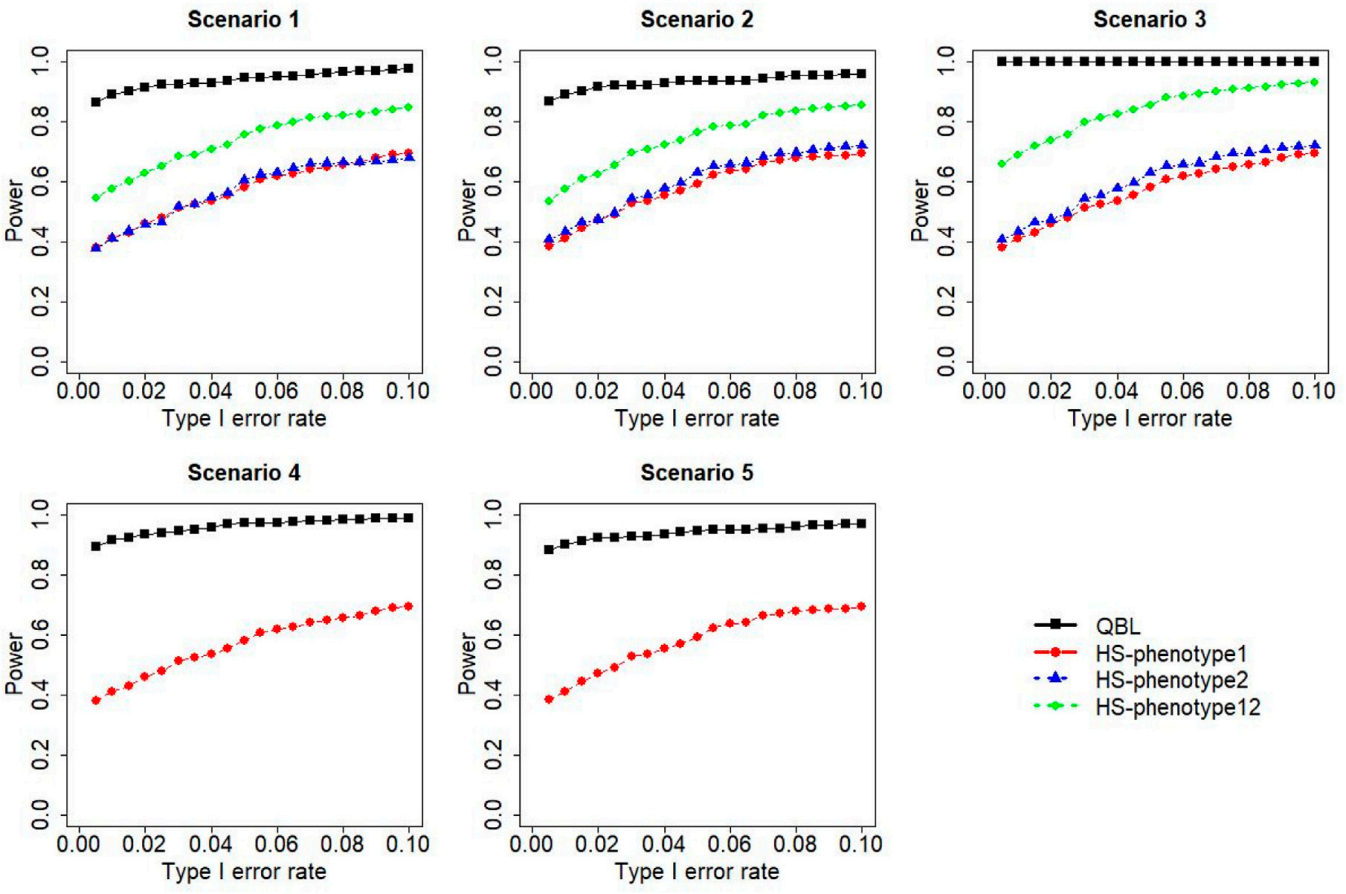
Simulation results under sample size 1,000, setting 2 (12 haplotypes), and *ρ* = 0.5. Scenarios are shown in Table 1. HS, Haplo.score; phenotype12: phenotype 1 or 2.

**FIGURE 12:**
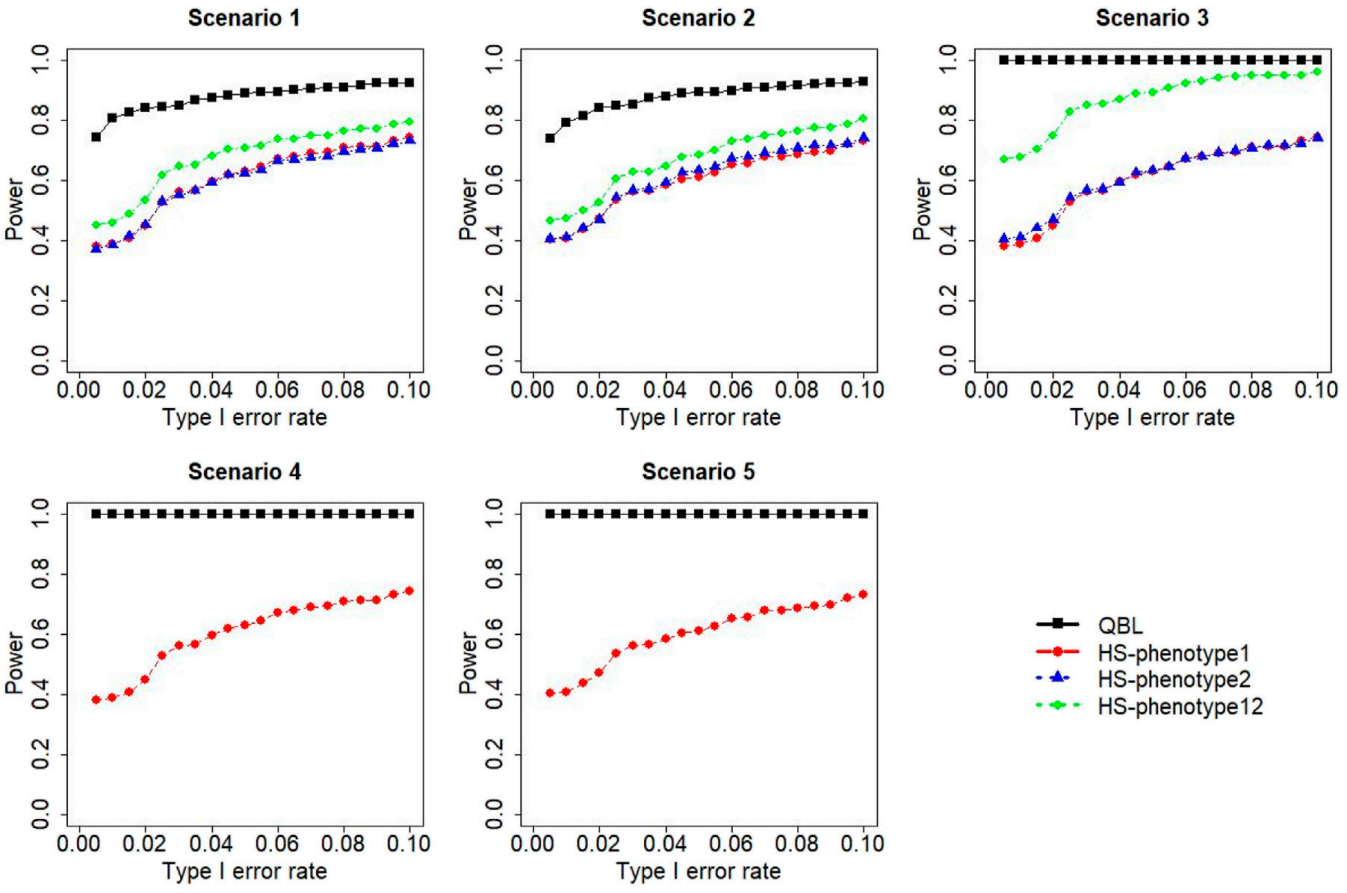
Simulation results under sample size 1,000, setting 2 (12 haplotypes), and *ρ* = 0.9. Scenarios are shown in Table 1. HS, Haplo.score; phenotype12, phenotype 1 or 2.

The results for settings 1 (six haplotypes) and 2 (12 haplotypes), sample sizes 500 and 1,000, and correlation coefficients 0.1, 0.5, and 0.9 are shown in Figures 1–12. Notably, bivariate QBL outperforms Haplo.score in all figures even though the margin of difference varies depending on the combination of association scenarios and ρ -values. Bivariate QBL shows the best performance in scenario 3, where the effect sizes of the target haplotype are in opposite directions (one β positive and another β negative). In this scenario, the power of bivariate QBL exceeds Haplo.score by a substantial margin. This margin increases in favor of QBL as the correlation coefficient increases. Bivariate QBL also maintains this superior performance in scenarios 4 and 5, where the target haplotype is unrelated to one phenotype but has a positive (scenario 4) or negative (scenario 5) association with the other phenotypes. Again, the power gain margin of bivariate QBL increases as the correlation between the two phenotypes increases. This outperformance trend can be seen in all combinations of haplotype settings and sample sizes considered in this study.

The performances of bivariate QBL and Haplo.score are the closest in the first two scenarios only when the correlation coefficient is high, i.e., 0.9, as shown in Figures 3, 6, 9. However, Figure 12 shows that even with ρ=0.9, bivariate QBL is clearly much more powerful than Haplo.score in these two scenarios. Moreover, when the correlation between the two phenotypes is weak or moderate, bivariate QBL outperforms Haplo.score in these scenarios at any combination of haplotype setting and sample sizes.

## 4 Application to GAW 19 data

We consider two continuous phenotypes, SBP and DBP, available in these data. They are moderately correlated (sample correlation coefficient = 0.55) and likely share a common genetic mechanism (Schillert and Konigorski, 2016). Typically, SBP and DBP are combined to create a single binary phenotype referred to as hypertension. More specifically, clinical thresholds are used for each BP to classify it as high blood pressure (BP); a subject is a case of hypertension if one of them is high (Datta and Biswas, 2016). However, converting a quantitative phenotype to a binary phenotype leads to a loss of information. Furthermore, combining them into one binary phenotype is a lost opportunity to investigate pleiotropy. As bivariate QBL can analyze the two continuous phenotypes jointly, it can potentially provide additional insight into these data.

There are 1,851 subjects in these data after discarding the missing values. Following Yuan and Biswas (2019), we analyze eight genes, namely, *FBN3*, *HRH1*, *INMT*, *MAP4*, *SAT2*, *SHBG*, *ULK4*, and *ZNF280D*. There are 28 SNVs in *FBN3*, 10 in *HRH1*, 18 in *INMT*, 18 in *MAP4*, 7 in *SAT2*, 15 in *SHBG*, 70 in *ULK4*, and 30 in *ZNF280D*. We combine five successive SNVs, starting from the first SNV, and create sliding haplotype blocks covering the whole gene, that is, on each gene, the first haplotype block consists of SNVs 1–5, second block consists of SNVs 2–6, and so on. For example, *ULK4* has 66 haplotype blocks and *MAP4* has 14 blocks.

We apply bivariate QBL to each haplotype block with both phenotypes jointly and Haplo.score to the same haplotype block twice with SBP and DBP separately. We calculate appropriate (and more general purpose) cutoffs for bivariate QBL and Haplo.score based on both simulated data and permutating the GAW19 phenotypes, as described in the following. We simulate 1,200 null samples, following setting 2 of Table 1. To match the GAW19 data more closely, we generate sample sizes of 1,851 with the correlation coefficient (between SBP and DBP) set to 0.55. As GAW19 data are exome sequence and have far more rare haplotypes than those considered in our simulations, we complement 1,200 simulated null samples by GAW19 data with permutated phenotype values. In particular, we permute the phenotypes of all subjects while retaining the pairing between SBP and DBP. Then, we combine the permuted phenotypes with genotypes in the *ULK4* gene to create a null sample. We repeat this process 10 times to obtain 660 (66 × 10) blocks or null samples. Similarly, the permuted phenotypes are also combined with genotypes from *MAP4* gene and repeated 10 times to provide 140 (14 × 10) blocks or null samples. The results from 800 null samples obtained using permutations are combined with those from 1,200 simulated null samples to calculate cutoffs.

The cutoffs based on 2000 null samples are calculated in the same manner, as described in the simulation study section for both bivariate QBL and Haplo.score. The cutoffs for type I error rates of 1% and 2.5% are found to be BFs of 10.91 and 4.65 for bivariate QBL and *p*-values of 0.0004 and 0.0058 for the Haplo.score global test, respectively.

The haplotype blocks found to be significantly associated at a type I error rate of 2.5% using at least one of the methods are shown in Table 2. Bivariate QBL found a larger number of haplotype blocks to be significant, and the findings are consistent with the literature (Datta et al., 2016; Yuan and Biswas, 2019). For example, Haplo.score could not detect the haplotype in *FBN3*, whose $\hat{\beta }$ values for SBP and DBP are in opposite directions. All the haplotype blocks found to be significant using Haplo.score are also detected by bivariate QBL. At the type I error rate of 1%, bivariate QBL identifies all haplotype blocks in *ULK4*, as shown in Table 2, as significant, whereas Haplo.score identifies only one haplotype block (39–43) as significant. Therefore, bivariate QBL appears to perform better than Haplo.score in GAW19 data, which is in agreement with our findings in the simulation study.

**TABLE 2:** Haplotype blocks significant at the 2.5% level on *ULK4*, *MAP4*, and *FBN3* genes using the bivariate QBL or Haplo.score (significant BF or *p*-value is shown in boldface).

				Bivariate QBL			Haplo.score	
Gene	Win	Hap	Freq	β (SBP)	β (DBP)	BF	*p*-value (SBP)	*p*-value (DBP)
*ULK4*	3–7	h10101	0.0016	1.206	0.824	**14.06**	0.0292	0.0913
*ULK4*	4–8	h01010	0.0014	1.608	0.747	**54.56**	**0.0056**	0.1308
*ULK4*	5–9	h10101	0.0014	1.619	0.767	**50.52**	**0.0033**	0.1319
*ULK4*	6–10	h01010	0.0016	1.211	0.843	**15.67**	**0.0011**	0.0405
*ULK4*	7–11	h10100	0.0016	1.218	0.849	**16.63**	**0.0007**	0.0335
*ULK4*	8–12	h01000	0.0016	1.207	0.836	**14.82**	**0.0009**	0.0477
*ULK4*	9–13	h10000	0.0017	1.209	0.835	**20.66**	**0.0012**	0.0384
*ULK4*	39–43	h11100	0.0055	0.869	0.666	**41.33**	**0.0001**	0.2726
*ULK4*	40–44	h11000	0.0052	0.854	0.801	**25.26**	0.0791	0.2656
*MAP4*	11–15	h10000	0.0043	0.778	1.714	**10.49**	0.0301	0.7634
*FBN3*	24–28	h00010	0.0014	0.783	−0.54	**10.41**	0.0313	0.2224

Win, window; Hap, haplotype; Freq, haplotype frequency.

## 5 Discussion

Health-related studies usually collect multiple outcomes to better assess patients’ health, understand complex diseases/traits, and inter-connection between them, which, in turn, can help in developing effective prevention and treatment strategies. These outcomes are often correlated and may share a common genetic etiology. A commonly used practice in genetic association studies is to analyze these outcomes in a one-at-a-time manner. Such a univariate approach essentially ignores the additional information contained in the joint distribution of the outcomes. Also, it is a missed chance to investigate the possibility of pleiotropy among these outcomes. Therefore, it is statistically and biologically more beneficial to adopt a multivariate approach to analyze the outcomes jointly. Moreover, analyzing haplotypes as genetic variants is advantageous because they are biologically interpretable, and haplotype-based tests can be performed on both NGS and GWAS data. There is no haplotype-based association test available that can detect rare variants associated with multiple continuous phenotypes yet. To fill this void, we propose bivariate QBL to detect the association of two quantitative traits with rare (and common) haplotypes. Our findings from the simulation study show that the method performs better than Haplo.score in all simulation setups that we considered.

Bivariate QBL performs best when the two outcomes have high positive correlation between them, and the target haplotype has discordant effects on the two phenotypes, i.e., one positive β and another negative β . This finding is consistent with the literature (Liu et al., 2009a; Ferreira and Purcell, 2009; Galesloot et al., 2014). In particular, to compare with Galesloot et al. (2014), we note that the first two scenarios in our study (both β s of the same sign) correspond to positive genetic correlation in their terminology, scenario 3 (one positive β and another negative β ) corresponds to negative genetic correlation, and scenarios 4 and 5 (one β is 0) correspond to no genetic correlation. In scenarios 3–5, with a negative or zero genetic correlation, bivariate QBL outperforms Haplo.score at any combination of haplotype settings, correlation, and sample sizes, and its power increases as the positive residual correlation (i.e., ρ in our context) increases. Bivariate QBL gains substantial power in these scenarios with increasing residual correlation as it not only avoids the burden of multiple testing but also incorporates the additional information provided by the cross-trait correlation. However, even with type I error rates of less than 1%, bivariate QBL has power close to or practically 1, whereas Haplo.score has a much lower power in these scenarios.

The performance of Haplo.score is close to that of bivariate QBL only when both outcomes are highly correlated and the target haplotype affects both outcomes in the same direction, i.e., scenarios 1 and 2. In these scenarios, the power of bivariate QBL increases as the correlation decreases. In the terminology of Galesloot et al. (2014), this means when both genetic correlation and residual correlation are of the same sign, the power of bivariate QBL decreases as the positive residual correlation increases. This phenomenon of bivariate QBL is also consistent with other multivariate genetic association tests that exist in the literature (Liu et al., 2009a; Ferreira and Purcell, 2009). In practice, it is unlikely that two phenotypes will have a very high correlation. On the other hand, we note that bivariate QBL estimates haplotype frequencies (***f***) jointly with the haplotype effects and other parameters. Haplotype frequencies are estimated very well by bivariate QBL, especially due to the fact that we set the starting values of ***f*** in the MCMC algorithm to its maximum likelihood estimate (obtained from the hapassoc package) (Burkett et al., 2006; Burkett et al., 2015). Thus, there is practically no impact of haplotype frequency estimation on type I error and power of the method.

In GAW19 data, SBP and DBP are moderately correlated (0.55) (Datta et al., 2016; Yuan and Biswas, 2019). As another example, Liu et al. (2009b) observed a correlation between the body mass index and bone mineral density of 0.384 and 0.257, respectively, in two datasets. When there is a weak-to-moderate correlation, bivariate QBL outperforms Haplo.score by a substantial margin. In our GAW19 data application, we detected several rare haplotype blocks to be associated with SBP and DBP jointly. Specifically, nine blocks were detected in *ULK4*, one in *MAP4*, and another in *FBN3*. These results agree with the findings from previous studies (Levy et al., 2009; International Consortium for Blood Pressure Genome-Wide Association Studies Ehret et al., 2011; Ehret and Caulfield, 2013). Notably, the correlation between SBP and DBP is moderate and as per our simulation results, bivariate QBL is far more powerful than Haplo.score in this situation. However, many of those haplotype blocks could not be detected by Haplo.score. This indicates that bivariate QBL can help establish multiple trait–variant associations and identify potential pleiotropic effects for further investigation.

Bivariate QBL has a limitation in terms of computing time. In our simulation study, for a sample size of 500, bivariate QBL takes 86 and 166 s to finish 2,00,000 MCMC iterations for 6 and 12 haplotypes, respectively. This is for a machine with 3.50-GHz Milan processor with 128 cores under the Linux operating system and 256 GB RAM. However, it is faster than both bivariate LBL-2B and LBL-BC. Bivariate QBL can handle a larger number of SNPs in a haplotype at the expense of an increased computational burden. The runtime of bivariate QBL almost doubles when we increase the number of SNPs in a haplotype block from 5 (86 s) to 10 (158 s). Another limitation is that the method can only accommodate two continuous phenotypes at a time. We plan to extend the framework of bivariate QBL (and LBL) to accommodate many correlated continuous and/or binary phenotypes jointly. We also plan to extend the framework to investigate gene–environment interactions and develop a computationally efficient version of this method.

Despite these limitations, we believe bivariate QBL is an important addition to the existing genetic association tests, especially because there is currently no rare haplotype association test available that can analyze two correlated continuous phenotypes jointly.

## 6 Software

An R package implementing the proposed bivariate QBL method will be made available at https://www.utdallas.edu/∼swati.biswas/ and https://github.com/ihsajal/ as part of the existing package LBL.

## Data Availability

The data analyzed in this study are subject to the following licenses/restrictions: The data are from Genetic Analysis Workshop 19. Participants of the workshop have access to these de-identified data for secondary analysis. Requests to access these datasets should be directed at https://bmcproc.biomedcentral.com/articles/10.1186/s12919-016-0007-z.
