# Supplementary material for: Bivariate quantitative Bayesian LASSO for detecting association of rare haplotypes with two correlated continuous phenotypes
Source: Front Genet. 2023 Mar 9;14:1104727. doi: 10.3389/fgene.2023.1104727 (PMC10033866; doi:10.3389/fgene.2023.1104727)
Supplement: Supplementary file 1 [file Presentation1.pdf]

Appendix

A1. MCMC steps

Given the parameter estimates at the tth iteration (denoted by upperscript t), we sample the new parameter values at the $(t+1)^{th}$ iteration in the following way.

Updating β_{0c}

We are using a normal prior for intercept β_{0c} with mean 0 variance 1. Let us denote $\boldsymbol{\beta_{-0c}}$ to be a vector of regression coefficient with element β_{jc} $(j = 1, 2, \dots, m-1)$. The conditional posterior distribution of β_{0c} is

$$\pi\left(\beta_{0c} \middle| \boldsymbol{\beta_{-0c}}, \boldsymbol{\beta_{c'}}, \sigma_c^2, \sigma_{c'}^2, \boldsymbol{u}, \sigma_u^2, \boldsymbol{f}, d, \boldsymbol{Y_c}, \boldsymbol{Y_{c'}}, \boldsymbol{Z}, \boldsymbol{G}\right)$$

$$\propto \pi(\beta_{0c}) \prod_{i=1}^{n} \sum_{z_{ir} \in S(G_i)} P(Y_{ic} |\, Z_{ir}, u_i) P(Y_{ic'} |\, Z_{ir}, u_i) P(\, Z_{ir}\,) P(u_i)$$

$$\propto \pi(\beta_{0c}) \prod_{i=1}^{n} \sum_{z_{ir} \in S(G_i)} P(Y_{ic} |\, Z_{ir}, u_i) P(Y_{ic'} |\, Z_{ir}, u_i) P(\, Z_{ir}\,)$$

Here, $P(Y_{ic} |\, Z_{ir}, u_i) \propto exp\left(\frac{(y_{ic}-u_i)\boldsymbol{X_{z_i}\beta_c}}{\sigma_c^2} - \frac{\left(\boldsymbol{X_{z_i}\beta_c}\right)^2}{2\sigma_c^2}\right)$.

Similarly, $P(Y_{ic'} |\, Z_{ir}, u_i) \propto exp\left(\frac{(y_{ic'}-u_i)\boldsymbol{X_{z_i}\beta_{c'}}}{\sigma_{c'}^2} - \frac{\left(\boldsymbol{X_{z_i}\beta_{c'}}\right)^2}{2\sigma_{c'}^2}\right)$.

$$\pi\left(\beta_{0c} \middle| \boldsymbol{\beta_{-0c}}, \boldsymbol{\beta_{c'}}, \sigma_c^2, \sigma_{c'}^2, \boldsymbol{u}, \sigma_u^2, \boldsymbol{f}, d, \boldsymbol{Y_c}, \boldsymbol{Y_{c'}}, \boldsymbol{Z}, \boldsymbol{G}\right)$$

$$\propto \frac{1}{2\pi} \exp\,(-\frac{\beta_{0c}^2}{2}) \prod_{i=1}^{n} \sum_{z_{ir} \in S(G_i)} P(Y_{ic} |\, Z_{ir}, u_i) P(Y_{ic'} |\, Z_{ir}, u_i) P(\, Z_{ir}\,)$$

$$\propto exp\left(-\frac{\beta_{0c}^2}{2}\right) \prod_{i=1}^{n} \sum_{z_{ir} \in S(G_i)} exp\left(\frac{(y_{ic}-u_i)\boldsymbol{X_{z_i}\beta_c}}{\sigma_c^2} - \frac{\left(\boldsymbol{X_{z_i}\beta_c}\right)^2}{2\sigma_c^2}\right)$$

$$exp\left(\frac{(y_{ic'}-u_i)\boldsymbol{X_{z_i}\beta_{c'}}}{\sigma_{c'}^2} - \frac{\left(\boldsymbol{X_{z_i}\beta_{c'}}\right)^2}{2\sigma_{c'}^2}\right) P(\, Z_{ir}\,|u_i) = g(\beta_{0c}), -\infty < \beta_{0c} < \infty.$$

β_{0c} is updated using Metropolis Hastings algorithm with proposal distribution $f\left(\beta_{0c}^{(*)} \middle| \beta_{0c}^{(t)}\right) = \mathrm{N}(\beta_{0c}^{(t)}, 1)$. $\beta_{0c}^{(t)}$ is the value of β_{0c} obtained in the tth MCMC iteration. The acceptance probability of the proposed move is $min\left(1, \frac{g(\beta_{0c}^{(*)}) f\left(\beta_{0c}^{(t)} \middle| \beta_{0c}^{(*)}\right)}{g(\beta_{0c}^{(t)}) f\left(\beta_{0c}^{(*)} \middle| \beta_{0c}^{(t)}\right)}\right)$.

Updating $\beta_{0c'}$

This is updated in the same manner as β_{0c} using $\mathrm{N}(\beta_{0c'}^{(t)}, 1)$ as the proposal distribution.

Updating β_{jc}

We update each element of $\boldsymbol{\beta_c}$ (β_{jc}) sequentially. The conditional posterior distribution of β_{jc} ($j = 1, 2, \dots, m-1$) is

$$\pi\left(\beta_{jc} \middle| \boldsymbol{\beta_{-jc}}, \boldsymbol{\beta_{c'}}, \sigma_c^2, \sigma_{c'}^2, \boldsymbol{u}, \sigma_u^2, \boldsymbol{f}, d, \boldsymbol{Y_c}, \boldsymbol{Y_{c'}}, \boldsymbol{Z}, \boldsymbol{G}\right)$$

$$\propto \pi(\beta_{jc}|\lambda_c) \prod_{i=1}^{n} \sum_{z_{ir} \in S(G_i)} P(Y_{ic}|\, Z_{ir}, u_i) P(Y_{ic'}|\, Z_{ir}, u_i) P(\, Z_{ir}\,) P(u_i)$$

$$\propto exp\left(-\lambda_c |\beta_{jc}|\right) \prod_{i=1}^{n} \sum_{z_{ir} \in S(G_i)} exp\left(\frac{(y_{ic} - u_i)\boldsymbol{X_{z_i}\beta_c}}{\sigma_c^2} - \frac{\left(\boldsymbol{X_{z_i}\beta_c}\right)^2}{2\sigma_c^2}\right)$$

$$exp\left(\frac{(y_{ic'} - u_i)\boldsymbol{X_{z_i}\beta_{c'}}}{\sigma_{c'}^2} - \frac{\left(\boldsymbol{X_{z_i}\beta_{c'}}\right)^2}{2\sigma_{c'}^2}\right) P(\, Z_{ir}\, |u_i)$$

$$= g\left(\beta_{jc}\right), -\infty < \beta_{jc} < \infty, j = 1, 2, \dots, m-1$$

Each element β_{jc} ($j = 1,2, \dots, m-1$) of $\boldsymbol{\beta_c}$ is updated using Metropolis Hastings algorithm with proposal distribution $f\left(\beta_{jc}^{(*)} \middle| \beta_{jc}^{(t)}\right)$ being double exponential with mean $\beta_{jc}^{(t)}$ and variance $|\beta_{jc}^{(t)}|$. $\beta_{jc}^{(t)}$ is the value of β_{jc} obtained in the $(t-1)$ MCMC iteration. The acceptance probability of the proposed move is $min\left(1, \frac{g(\beta_{jc}^{(*)}) f\left(\beta_{jc}^{(t)} \middle| \beta_{jc}^{(*)}\right)}{g(\beta_{jc}^{(t)}) f\left(\beta_{jc}^{(*)} \middle| \beta_{jc}^{(t)}\right)}\right)$.

Updating $\beta_{jc'}$

This is updated in the same manner as β_{jc} using double exponential proposal with mean $\beta_{jc'}^{(t)}$ and variance $|\beta_{jc'}^{(t)}|$.

Updating λ_c and $\lambda_{c'}$

The conditional posterior distribution of λ_c is

$\pi(\lambda_c|\boldsymbol{\beta_c}) \propto \pi(\lambda_c)\pi(\boldsymbol{\beta_c}|\lambda_c)$

$$\propto \lambda_c^{a-1} exp(-\lambda_c b) \prod_{j=1}^{m-1} \frac{\lambda_c}{2} exp\left(-\lambda_c\left|\beta_{jc}\right|\right) \propto \lambda_c^{m+a-1} exp\left[-\lambda_c\left(b+\sum_{j=1}^{m-1}\left|\beta_{jc}\right|\right)\right]$$

This is proportional to Gamma distribution with shape parameter $(m+a)$ and rate parameter $\left(b+\sum_{j=1}^{m-1}\left|\beta_{jc}\right|\right)$. We can use Gibbs sampler to directly sample λ_c from the derived conditional posterior distribution. Similarly, we can sample $\lambda_{c'}$ from the conditional posterior distribution Gamma with shape and rate parameters being $(m+a)$ and $\left(b+\sum_{j=1}^{m-1}\left|\beta_{jc'}\right|\right)$, respectively.

Updating $\boldsymbol{f}$

The conditional posterior distribution of $\boldsymbol{f}$ is

$\pi\left(\boldsymbol{f}\middle|\boldsymbol{\beta_c}, \boldsymbol{\beta_{c'}}, \sigma_c^2, \sigma_{c'}^2, \boldsymbol{u}, \sigma_u^2, \boldsymbol{Y_c}, \boldsymbol{Y_{c'}}, \boldsymbol{Z}, \boldsymbol{G}\right)$

$$\propto \pi(d|\boldsymbol{f})\pi(\boldsymbol{f}) \prod_{i=1}^{n} \sum_{z_{ir}\in S(G_i)} P(Y_{ic}|\, Z_{ir}\,, u_i) P(Y_{ic'}|\, Z_{ir}\,, u_i) P(\, Z_{ir}\,) P(u_i)$$

$$\propto \frac{I\left(\max_k\left\{-\frac{f_k}{1-f_k}\right\} < d < 1\right)}{1-\max_k\left\{-\frac{f_k}{1-f_k}\right\}} \prod_{i=1}^{n} \sum_{z_{ir}\in S(G_i)} P(Y_{ic}|\, Z_{ir}\,, u_i) P(Y_{ic'}|\, Z_{ir}\,, u_i)\, P(\, Z_{ir}\,) = g(\boldsymbol{f}).$$

$\boldsymbol{f}$ is updated using Metropolis Hastings algorithm with proposal distribution $f(\boldsymbol{f}^{(*)}|\boldsymbol{f}^{(t)})$ being Dirichlet$(a_1, a_2, \dots, a_m)$, where $f_1^{(t)} = \frac{a_1}{a_0}$, $f_2^{(t)} = \frac{a_2}{a_0}$, …, $f_m^{(t)} = \frac{a_m}{a_0}$, $a_0 = \sum_{i=1}^{m} a_i = C$. The acceptance probability of the proposed move is $min\left(1, \frac{g(\boldsymbol{f}^{(*)})f(\boldsymbol{f}^{(t)}|\boldsymbol{f}^{(*)})}{g(\boldsymbol{f}^{(t)})f(\boldsymbol{f}^{(*)}|\boldsymbol{f}^{(t)})}\right)$.

We use the R package “hapassoc” to obtain the initial estimates of the frequencies of *m* haplotypes and use those as the starting values in the MCMC algorithm.

Updating d

The conditional posterior distribution of d is

$$\pi\left(d \middle| \boldsymbol{\beta_c}, \boldsymbol{\beta_{c'}}, \sigma_c^2, \sigma_{c'}^2, \boldsymbol{f}, \boldsymbol{u}, \sigma_u^2, \boldsymbol{Y_c}, \boldsymbol{Y_{c'}}, \boldsymbol{Z}, \boldsymbol{G}\right)$$

$$\propto \pi(d|\boldsymbol{f}) \prod_{i=1}^{n} \sum_{z_{ir} \in S(G_i)} P(Y_{ic}|\, Z_{ir}, u_i) P(Y_{ic'}|\, Z_{ir}, u_i) P(\, Z_{ir}\,) P(u_i)$$

$$\propto I\left(\max_k\left\{-\frac{f_k}{1-f_k}\right\} < d < 1\right) \prod_{i=1}^{n} \sum_{z_{ir} \in S(G_i)} P(Y_{ic}|\, Z_{ir}, u_i) P(Y_{ic'}|\, Z_{ir}, u_i) P(\, Z_{ir}\,) = g(d)$$

Here, d is updated using Metropolis Hastings algorithm with proposal distribution $f(d^{(*)}|d^{(t)})$ being Uniform$(d^{(t)} - \nu, d^{(t)} + \nu)$, where $\nu = 0.05$. The constraint $\max_k\left\{-\frac{f_k}{1-f_k}\right\} < d^{(t+1)} < 1, k = 1, 2, \dots, m$ needs to be satisfied at each update. The acceptance probability of the proposed move is $min\left(1, \frac{g(d^{(*)})f(d^{(t)}|d^{(*)})}{g(d^{(t)})f(d^{(*)}|d^{(t)})}\right)$.

Updating $\boldsymbol{u}$

The conditional posterior distribution of u_i is

$$\pi\left(u_i \middle| \boldsymbol{\beta_c}, \boldsymbol{\beta_{c'}}, \sigma_c^2, \sigma_{c'}^2, \boldsymbol{f}, \sigma_u^2, \boldsymbol{Y_c}, \boldsymbol{Y_{c'}}, \boldsymbol{Z}, \boldsymbol{G}\right) \propto p(u_i) \sum_{z_{ir} \in S(G_i)} P(Y_{ic}|\, Z_{ir}, u_i) P(Y_{ic'}|\, Z_{ir}, u_i) P(\, Z_{ir}\,)$$

$$\propto exp\left(\frac{u_i^2}{2\sigma_u^2}\right)\sum_{z_{ir}\in S(G_i)} exp\left(\frac{(y_{ic}-u_i)\boldsymbol{X}_{\boldsymbol{z}_i}\boldsymbol{\beta}_{\boldsymbol{c}}}{\sigma_c^2}-\frac{\left(\boldsymbol{X}_{\boldsymbol{z}_i}\boldsymbol{\beta}_{\boldsymbol{c}}\right)^2}{2\sigma_c^2}\right) exp\left(\frac{(y_{ic'}-u_i)\boldsymbol{X}_{\boldsymbol{z}_i}\boldsymbol{\beta}_{\boldsymbol{c}'}}{\sigma_{c'}^2}\right.$$

$$\left.-\frac{\left(\boldsymbol{X}_{\boldsymbol{z}_i}\boldsymbol{\beta}_{\boldsymbol{c}'}\right)^2}{2\sigma_{c'}^2}\right) P(\,Z_{ir}\,|u_i)$$

$$= g(u_i), i = 1, 2, \dots, n$$

Here, each u_i is updated using Metropolis Hastings algorithm with proposal distribution $f(u_i^{(*)}|u_i^{(t)})$ being Normal$(u_i^{(t)}, |{u_i}^{(t)}|)$. The acceptance probability of the proposed move is $min\left(1, \frac{g(u_i^{(*)})f(u_i^{(t)}|u_i^{(*)})}{g(u_i^{(t)})f(u_i^{(*)}|u_i^{(t)})}\right)$.

Updating σ_u

$\sigma_u^{(*)}$ is generated from the proposal distribution $f(\sigma_u^{(*)}|\sigma_u^{(t)})$ that is Normal$\left(\sigma_u^{(t)}, \left(\frac{\sigma_u^{(t)}}{20}\right)^2\right)$. Here, if $\sigma_u^{(*)} \leq 0$, we set $\sigma_u^{(t+1)} = \sigma_u^{(t)}$. Otherwise, σ_u is updated using Metropolis Hastings algorithm. The conditional posterior distribution of σ_u is

$$\pi(\sigma_u|\boldsymbol{u}) \propto \pi(\sigma_u)\prod_{i=1}^{n} p(u_i) \propto \left(\frac{1}{1+\left(\frac{\sigma_u}{A}\right)^2}\right)\prod_{i=1}^{n}\frac{1}{\sigma_u} exp\left(-\frac{u_i^2}{2\sigma_u^2}\right)$$

$$= \left(\frac{1}{\sigma_u}\right)^n \left(\frac{1}{1+\left(\frac{\sigma_u}{A}\right)^2}\right) exp\left(-\frac{\sum_{i=1}^{n} u_i^2}{2\sigma_u^2}\right) = g(\sigma_u).$$

The acceptance probability of the proposed move is $min\left(1, \frac{g(\sigma_u^{(*)})f(\sigma_u^{(t)}|\sigma_u^{(*)})}{g(\sigma_u^{(t)})f(\sigma_u^{(*)}|\sigma_u^{(t)})}\right)$.

Updating σ_c^2

The conditional posterior distribution of σ_c^2 is

$$\pi\left(\sigma_c^2\middle|\boldsymbol{\beta}_{\boldsymbol{c}}, \boldsymbol{\beta}_{\boldsymbol{c}'}, \sigma_{c'}^2, \boldsymbol{u}, \sigma_u^2, \boldsymbol{f}, d, \boldsymbol{Y}_{\boldsymbol{c}}, \boldsymbol{Y}_{\boldsymbol{c}'}, \boldsymbol{Z}, \boldsymbol{G}\right)$$

$$\propto \prod_{i=1}^{n} \sum_{z_{ir} \in S(G_i)} \pi(\sigma_c^2) P(Y_{ic} | Z_{ir}, u_i) P(Y_{ic'} | Z_{ir}, u_i) P(Z_{ir})$$

$$\propto \left(-\frac{1}{\sigma_c^2}\right)^{\frac{n+1}{2}} \prod_{i=1}^{n} \sum_{z_{ir} \in S(G_i)} exp\left(\frac{(y_{ic} - u_i)\boldsymbol{X_{z_i}\beta_c}}{\sigma_c^2} - \frac{\left(\boldsymbol{X_{z_i}\beta_c}\right)^2}{2\sigma_c^2}\right)$$

$$exp\left(\frac{(y_{ic'} - u_i)\boldsymbol{X_{z_i}\beta_{c'}}}{\sigma_{c'}^2} - \frac{\left(\boldsymbol{X_{z_i}\beta_{c'}}\right)^2}{2\sigma_{c'}^2}\right) P(Z_{ir})$$

$$= g(\sigma_c^2)$$

Here, ${\sigma_c^{(*)}}^2$ is generated from proposal distribution $f\left({\sigma_c^{(*)}}^2 | {\sigma_c^{(t)}}^2\right)$ being Normal$\left({\sigma_c^{(t)}}^2, \left(\frac{{\sigma_c^{(t)}}^2}{15}\right)^2\right)$.

If ${\sigma_c^{(*)}}^2 \leq 0$, we set ${\sigma_c^{(t+1)}}^2 = {\sigma_c^{(t)}}^2$. Otherwise, σ_c^2 is updated using Metropolis Hastings algorithm. The acceptance probability of the proposed move is $min\left(1, \frac{g\left({\sigma_c^{(*)}}^2\right) f\left({\sigma_c^{(t)}}^2 | {\sigma_c^{(*)}}^2\right)}{g\left({\sigma_c^{(t)}}^2\right) f\left({\sigma_c^{(*)}}^2 | {\sigma_c^{(t)}}^2\right)}\right)$.

Updating $\sigma_{c'}^2$

This is updated in the same manner as σ_c^2 using Normal$\left({\sigma_{c'}^{(t)}}^2, \left(\frac{{\sigma_{c'}^{(t)}}^2}{15}\right)^2\right)$ as the proposal.

A2. Prior odds calculation

The prior odds of H_a to H_0 can be obtained using the unconditional joint prior distribution of $(\beta_{jc}, \beta_{jc'})$.

Consider the marginal prior distribution of β given by

$$\pi(\beta) = \int_\lambda \pi(\beta|\lambda)\pi(\lambda) d\lambda$$

$$= \int_\lambda \frac{\lambda}{2} \exp(-\lambda|\beta|) \frac{b^a}{\Gamma(a)} \lambda^{a-1} exp(-\lambda b)\, d\lambda$$

$$= \frac{b^a}{2\Gamma(a)} \int_\lambda \lambda^{a-1+1} \exp(-\lambda(|\beta| + b)\, d\lambda = \frac{b^a}{2\Gamma(a)} \frac{\Gamma(a+1)}{(|\beta| + b)^{a+1}}$$

$$= \frac{ab^a}{2(|\beta| + b)^{a+1}}$$

Now, we can compute $P(|\beta| \leq \epsilon)$ as below

$$P(|\beta| \leq \epsilon) = \int_{-\epsilon}^{\epsilon} \frac{ab^a}{2(|\beta| + b)^{a+1}} d\beta = \frac{ab^a}{2} \left[\int_{-\epsilon}^{0} (|\beta| + b)^{-a-1} d\beta + \int_{0}^{\epsilon} (|\beta| + b)^{-a-1} d\beta \right]$$

$$= \frac{b^a}{2} [b^{-a} - (\epsilon + b)^{-a} - (\epsilon + b)^{-a} + b^{-a}] = 1 - \frac{b^a}{(\epsilon + b)^a}.$$

Hence, we get

$$P(|\beta| > \epsilon) = \frac{b^a}{(\epsilon + b)^a}.$$

Using independence of β_{jc} and $\beta_{jc'}$, we can compute the prior probability of H_0 as

$$P(|\beta_{jc}| \leq \epsilon \text{ and } |\beta_{jc'}| \leq \epsilon) = P(|\beta_{jc}| \leq \epsilon) P(|\beta_{jc'}| \leq \epsilon) = \left(1 - \frac{b^a}{(\epsilon + b)^a}\right)^2$$

$$= 1 - \frac{2b^a}{(\epsilon + b)^a} + \frac{b^{2a}}{(\epsilon + b)^{2a}}.$$

The prior probability of H_a is

$$P(|\beta_{jc}| > \epsilon \text{ or } |\beta_{jc'}| > \epsilon) = 1 - P(|\beta_{jc}| \leq \epsilon) P(|\beta_{jc'}| \leq \epsilon) = \frac{2b^a}{(\epsilon + b)^a} - \frac{b^{2a}}{(\epsilon + b)^{2a}}.$$

Therefore, the prior odds of H_a to H_0 is

$$\frac{P(|\beta_{jc}| > \epsilon \text{ or } |\beta_{jc'}| > \epsilon)}{P(|\beta_{jc}| \leq \epsilon \text{ and } |\beta_{jc'}| \leq \epsilon)} = \frac{(2b^a(\epsilon + b)^a - b^{2a})}{((\epsilon + b)^a - b^a)^2}; \quad -\infty < \beta_{jc}, \beta_{jc'} < \infty,$$

Setting $a = b = 20$ and $\epsilon = 0.1$ give the prior odds to be 109.95.
